# Development and validation of HPLC-FLD method for simultaneous determination of ternary therapy used for COVID-19 in plasma samples

**DOI:** 10.1038/s41598-026-53885-7

**Published:** 2026-06-16

**Authors:** Eman A. Madbouly, Abdalla A. El-Shanawani, Sobhy M. El-Adl, Ahmed S. Abdelkhalek

**Affiliations:** Department of Medicinal Chemistry, Faculty of Pharmacy, Zagazig University, Zagazig, Egypt

**Keywords:** Covid-19, Remdesivir, Moxifloxacin, Levodropropizine, Micellar HPLC, Fluorescence detection, Eco scale, GAPI, Green assessment, Chemistry, Drug discovery

## Abstract

A novel green sensitive micellar HPLC method with fluorescence detection is introduced for the analysis of remdesivir, levodropropizine, and moxifloxacin hydrochloride as ternary therapy for COVID-19 in human plasma samples using losartan as internal standard. The separation was carried out on Hypersil BDS C18 column using a mixed micellar mobile phase of sodium lauryl sulfate (SLS), Polyoxyethylene (23) lauryl ether (Brij-35) adjusted to pH 6. For enhanced specificity, a fluorescence detector was set to 246ex/403em for remdesivir, 296ex/504em for moxifloxacin hydrochloride , 252ex/342em for levodropropizine, and 252ex/316em for Losartan. The method showed a linear response within the concentration ranges of 80-2000 ng/mL for remdesivir and moxifloxacin hydrochloride and 160–4000 ng/mL for levodropropizine showing high accuracy and precision. The current study’s significance lies in the development of a highly sensitive and selective analytical method for the simultaneous determination of three different drugs (remdesivir, moxifloxacin hydrochloride, and levodropropizine) in spiked human plasma, using an economical sample preparation strategy. Eco scale, GAPI and AGREE confirmed the greenness of the method which shows the least impact on the environment along with high score for eco-friendliness. This creates a new approach for routine quality control and pharmacokinetic research of remdesivir, levodropropizine and moxifloxacin hydrochloride.

**Supplementary Information:**

The online version contains supplementary material available at 10.1038/s41598-026-53885-7.

## Introduction

COVID-19 is considered as the most significant public health emergency of the last century, characterized as a rapidly spreading respiratory infection^1^. The need for an effective oral antiviral medication persists due to low vaccine uptake, even with several immunizations administered since 2020 to combat SARS-COV-2. Approving new therapies is a lengthy process, so utilizing FDA-approved medications like remdesivir and favipiravir has been the most expedient approach^2,3^.

**Fig. 1:**
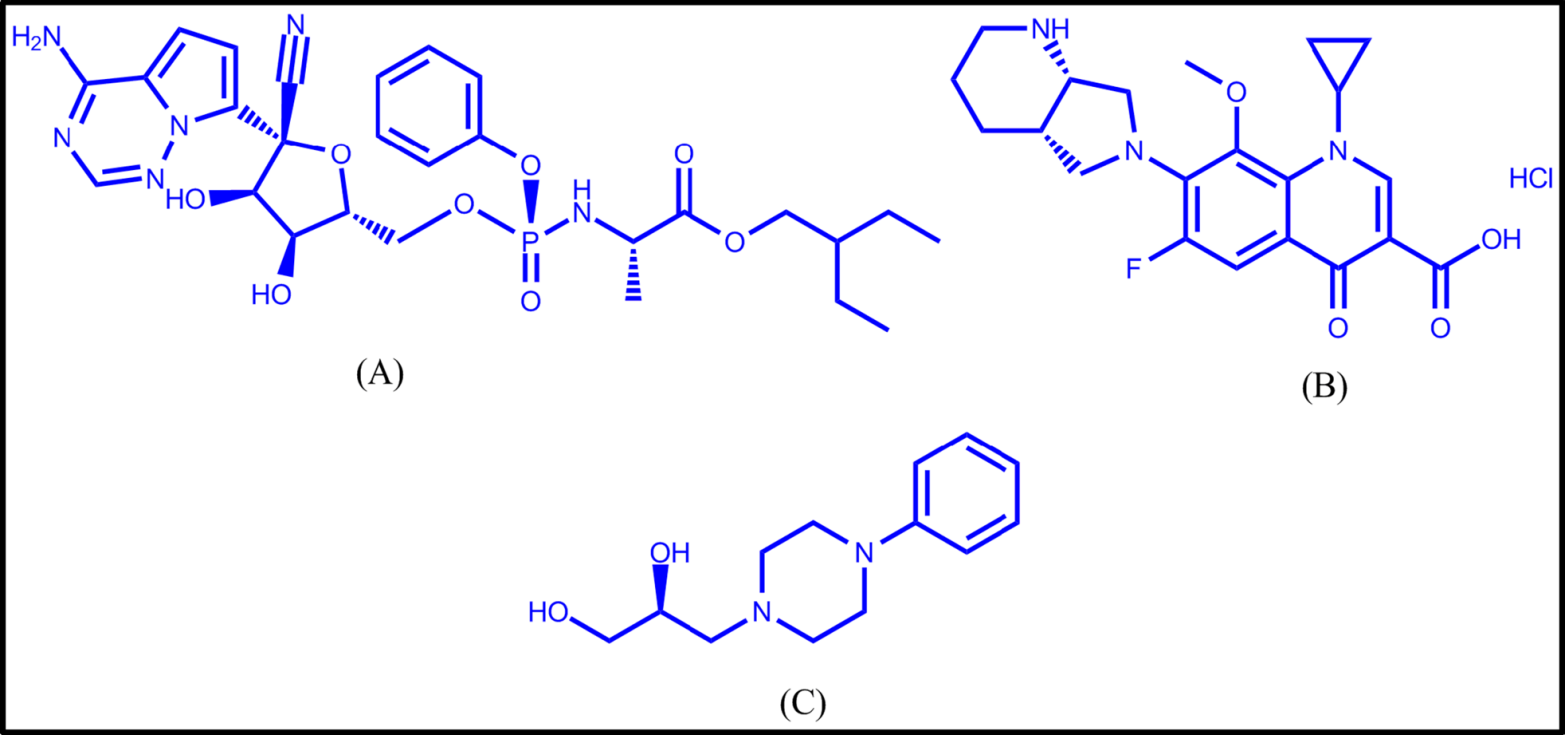
The chemical structure of RDV (**A**), MFX (**B**) and LDP (**C**).

Remdesivir (RDV) (Fig. 1a) is an adenosine triphosphate analog with broad antiviral activity, showing potential to expedite recovery in hospitalized COVID-19 patients. It was the first drug authorized by the US FDA for emergency use in treating such patients. RDV inhibits RNA polymerase, thereby preventing coronavirus replication^4,5^. RDV measurements have been published using a variety of approaches, including chromatography^6–9^, spectrophotometry^10–15^, electrochemistry^16,17^ and spectrofluorimetry^18–22^.

COVID-19, being a viral infection, presents challenges when treated with antibiotics, which do not affect SARS-CoV-2 directly. However, viral infections can heighten the risk of bacterial pneumonia^23^. A new in silico study suggests that fluoroquinolones like moxifloxacin hydrochloride (MFX), Fig. 1b, may inhibit SARS-CoV-2 replication through their strong binding to the virus’s main protease enzyme^24,25^. The literature review enumerates various analytical techniques for MFX analysis, encompassing densitometry^26,27^, chromatography^28–33^, spectrophotometry^34–36^, and spectrofluorometry^37–39^. While LC-MS/MS offers high sensitivity for plasma analysis^30,32^, simpler methods are more accessible but less suitable for trace-level detection.

Antitussive drugs, such as levodropropizine (LDP), demonstrated in Fig. 1c, are used to treat COVID-19 symptoms. LDP blocks the activation of vagal C fibers, which results in a highly strong antitussive effect that is mostly dependent on its peripheral activity, even though little is known about the chemical mechanism of action^40^. Numerous analytical techniques, such as chromatographic^41–44^, spectrophotometric^45,46^, electrochemical^47,48^, and spectroflourimetric^49,50^ procedures, have been previously developed for quantifying LDP. Similar to other methods, chromatographic approaches provide precision but involve high solvent consumption, whereas spectrophotometric and spectrofluorimetric techniques are simpler but less selective. The studied drugs are commonly co-administered in COVID-19 management protocols, where RDV acts as an antiviral agent, MFX is used to control secondary bacterial infections, and LDP serves as an antitussive. Therefore, their simultaneous determination is important for therapeutic monitoring and potential pharmacokinetic applications. Previously reported methods for RDV, MFX, and LDP were mainly based on HPLC with UV detection using organic solvents such as acetonitrile or methanol. These methods are limited in terms of environmental impact, sensitivity, and applicability to simultaneous multicomponent analysis in biological matrices.

The ability of chromatographic techniques, especially the RP-HPLC technology, to rapidly and precisely separate and quantify a variety of analyte combinations gives them an advantage over other approaches. On the other hand, research and pharmaceutical quality control laboratories do a staggering number of regular analyses every day. Because of their massive waste disposals, this might be an environmental issue. A single traditional HPLC system has the capacity to produce 0.5 L or more of organic waste every day^51^. Thus, in an effort to lessen the industrial influence on environmental contamination, ecologically safer analytical techniques ought to be developed in compliance with the green analytical chemistry tenets. One environmentally friendly tool for chromatographic procedures is micellar liquid chromatography^52^. The application of mixed micellar mobile phases resulted in reduced analyte retention times, improved separation efficiency in columns, and the elimination of the necessity for elution with organic solvents that pose a risk to the environment^53^. Sodium lauryl sulphate (SLS) and Brij-35, a non-ionic polyoxyethylene-23-lauryl ether surfactant, have become popular substitutes for organic solvents in recent years^8,54^. Nevertheless, HPLC could not have the sensitivity needed for bioanalytical uses. On the other hand, HPLC and fluorescence detection (FLD) provide an affordable alternative that greatly improves the detection of drug signals at considerably lower concentrations^55^.

Despite the variety of reported methods, no approach has been reported for the simultaneous determination of RDV, MFX, and LDP in plasma that combines high sensitivity, speed, and environmental compliance. Existing methods are limited either by solvent consumption, insufficient selectivity in multicomponent matrices, or lack of suitability for simultaneous analysis.

So, the present study developed a sensitive, rapid, and green HPLC-FLD method for simultaneous quantification of RDV, MFX, and LDP in plasma, with applications to pharmaceutical formulations.

The therapeutic efficacy of drugs is closely related to their plasma concentrations; therefore, accurate determination of drug levels in biological matrices is essential for pharmacokinetic investigations and therapeutic monitoring^56^. Accordingly, the proposed method was designed for the determination of the studied drugs in spiked human plasma. In addition, the developed method was successfully applied for the analysis of the investigated drugs in their pharmaceutical formulations for routine quality control purposes.

Losartan (LOS) was employed as an internal standard due to its suitable chromatographic behavior and physicochemical properties, which ensure adequate retention, stable fluorescence response under the optimized conditions, and comparable extraction recovery. Moreover, LOS does not interfere with the analyte peaks, allowing accurate correction for variability in sample preparation and instrument response^57,58^.

In addition, the environmental impact of the proposed analytical procedure was evaluated using the Analytical Eco-Scale and the Green Analytical Procedure Index (GAPI) tools. These greenness assessment approaches provide a comprehensive evaluation of solvent consumption, reagent hazards, and overall environmental impact, ensuring that the developed method aligns with the principles of green analytical chemistry^59,60^.

## Experimental

### Instruments

Chromatographic analysis was performed using a Waters Alliance 2695 HPLC separation module equipped with a quaternary pump, solvent cabinet, and autosampler injector (Waters Corporation, USA). Detection was carried out using a Waters 2475 fluorescence detector. The separation was achieved on a Hypersil BDS (Base Deactivated Silica) C18 column (150 × 4.6 mm, 5 μm particle size).

A bench pH meter (AD1030, ADWA, Romania) was used for pH adjustment, and a sonicator (WUC-A06H, Germany) was used for solution degassing. Data acquisition and chromatographic processing were performed using Empower 3 Chromatography Data Software (Version 3; Waters Corporation, Milford, MA, USA; https://www.waters.com/nextgen/us/en/products/informatics-and-software/chromatography-software/empower-software-solutions/empower-cds.html), while Microsoft Excel 2010 (Version 14.0; Microsoft Corporation, Redmond, WA, USA; https://www.microsoft.com/en-us/microsoft-365/excel) was used for calculations.

### Materials

All reagents were of analytical reagent grade. Sodium lauryl sulfate (SLS, ≥ 99%) and Brij-35 (≥ 99%) were obtained from Merck (Darmstadt, Germany), while methanol (HPLC grade) was also purchased from Merck (Darmstadt, Germany). Sodium dihydrogen phosphate (NaH₂PO₄) and sodium hydroxide were supplied by El-Nasr Pharmaceutical Chemicals Co. (Cairo, Egypt).

A generous donation of RDV powder (99.15%) and MFX (99.45%) pure powder was made by EVA Pharma (Cairo, Egypt). LDP powder (99.70%) was kindly provided by Atco Pharma (Menofia, Egypt). Plasma samples from Zagazig University Hospital (Zagazig, Egypt) were generously provided and stored at − 20 °C until analysis.

Remdesivir-Eva vials were acquired from the local market. This product is produced by EVA Pharma, located in Cairo, Egypt, and is associated with batch numbers 2,009,561, 2,009,563, 2,009,566, and 2,009,567. The label indicates a concentration of 100 mg per 20 mL of remdesivir. Tussistop Tablet (60 mg LDP/Tablet) manufactured by Atco Pharma for Pharmaceutical Industries (batch number 220811) was purchased on the local market. Moxiflox Tablet (400 mg MFX/Tablet), manufactured by EVA Pharmaceutical Industrial Company (batch number 2212537), was purchased locally. Losartan potassium (LOS) pure powder (99.70%) was provided by EIPICO (10th of Ramadan, Egypt) and used as internal standard (IS).

### Standard solutions

Stock standard solutions (1 mg mL⁻¹) of remdesivir (RDV), moxifloxacin hydrochloride (MFX), levodropropizine (LDP), and losartan (LOS) were prepared separately by dissolving 100 mg of each compound in about 70 mL methanol, followed by sonication for 15 min, and then the volume was completed to 100 mL with the mobile phase (MP).

Working standard solutions were prepared by appropriate dilution of the stock solutions with the mobile phase to obtain a final concentration of 10 µg mL⁻¹ for each analyte and the internal standard.

Stock standard solutions of RDV, MFX, LDP, and LOS were stored at **4 °C** and were stable for two weeks. Working solutions were freshly prepared daily before analysis. Spiked plasma samples were stored at **− 20 °C** until analysis.

## Procedures

### Chromatographic conditions

Chromatographic separation and HPLC-FLD analysis were performed on a Hypersil BDS (Base Deactivated Silica) C18 column (150 × 4.6 mm, 5 μm; Thermo Scientific) using an isocratic elution system. The mobile phase consisted of 0.1 mol L⁻¹ sodium lauryl sulfate (SLS), 0.03 mol L⁻¹ Brij-35, and 0.02 mol L⁻¹ sodium dihydrogen phosphate (NaH₂PO₄). The pH was adjusted to 6.0 ± 0.1 using orthophosphoric acid, and the buffer system provided adequate buffering capacity within the working range.

The mobile phase composition was optimized based on system suitability parameters to achieve acceptable peak symmetry, resolution, and efficiency for all analytes. The selected conditions (0.1 mol L⁻¹ SLS, 0.03 mol L⁻¹ Brij-35, and pH 6.0) provided the best compromise between retention behavior and chromatographic performance.

The flow rate was maintained at 1.0 mL min⁻¹, and the column temperature was kept at 40 °C. A sample injection volume of 50 µL was used to enhance detection sensitivity, particularly for low analyte concentrations in plasma samples.

Fluorescence detection was performed using a time-programmed mode with four excitation/emission wavelength pairs: 246/403 nm (RDV), 296/504 nm (MFX), 252/342 nm (LDP), and 252/316 nm (LOS).

Under the optimized chromatographic conditions, all analytes were baseline separated within a total run time of less than 12 min, with retention times of 6.1, 7.2, 9.2, and 3.2 min for RDV, MFX, LDP, and LOS, respectively.

### Calibration curves construction

Calibration standards were prepared in three independent sets using 10 mL volumetric flasks. Appropriate aliquots from the standard working solutions of each drug were accurately transferred along with a fixed volume of losartan (LOS) as an internal standard (IS). The solutions were then diluted to volume with the mobile phase (MP) to obtain final concentration ranges of 80–2000 ng mL⁻¹ for RDV and MFX, and 160–4000 ng mL⁻¹ for LDP, while maintaining a constant IS concentration of 2000 ng mL⁻¹.

Calibration curves were constructed using seven concentration levels for each analyte within the specified linearity ranges. Each calibration level was injected in triplicate (*n* = 3) using an injection volume of 50 µL, and the corresponding peak areas were recorded.

Calibration curves were obtained by plotting the peak area ratios (analyte/IS) against the corresponding analyte concentrations. An unweighted least-squares linear regression model was applied to establish the relationship between concentration and detector response for each analyte under the optimized chromatographic conditions.

The obtained data were statistically evaluated and expressed as mean values ± %RSD. The calibration model showed excellent linearity with correlation coefficients (r² ≥ 0.9997), and the back-calculated concentrations were within acceptable limits.

### Laboratory prepared mixtures analysis

Various aliquots of RDV, LDP, and MFX, each containing a specific volume of LOS, were transferred from their corresponding working standard solutions into 10-mL volumetric flasks as part of the current study. MP was then added to dilute the aliquots to their final concentrations. This led to the creation of combinations with different percentages of each drug that were within its linear range. Following that, the prepared solutions were auto-injected (50 µL) into the column and, as previously stated, chromatographed (in triplicate) under HPLC chromatographic conditions. Finally, the concentrations of the aforementioned medications were calculated using their corresponding regression equations.

### Application to pharmaceuticals

#### RDV

To prepare the solution, mix four vials of Remdesivir-Evapharma (100 mg / 20 mL) and add 0.2 mL of RDV (1 mg) to a 100 mL volumetric flask with a specified volume of LOS (IS) to obtain a final IS concentration of 2000 ng/mL. Add about 70 mL of methanol. After 15 min of vigorous shaking, the solution was sonicated for 30 min. To get to a concentration of 10 µg/mL, MP was added to 100 mL. The solution was then filtered through a 0.45 μm membrane filter before analysis.

#### MFX

Ten tablets of Moxiflox (436.37 mg of moxifloxacin HCl, equivalent to 400 mg of moxifloxacin per tablet) were weighed and ground into a coarse powder. A portion of the powder equivalent to 10 mg of MFX was precisely weighed and transferred to a 100 mL volumetric flask with a known volume of LOS (IS) to obtain a final IS concentration of 2000 ng/mL. About 70 mL of methanol was added and the mixture was shaken vigorously for 15 min, followed by sonication for 30 min to ensure complete extraction. The solution was then diluted to volume with the mobile phase (MP) to obtain a concentration of 100 µg/mL and filtered through a 0.45 μm membrane filter. Further dilution with MP produced a working solution of 10 µg/mL.

#### LDP

Ten Tussistop tablets (60 mg LDP/tablet) were weighed and ground into a coarse powder. A portion of the powder equivalent to 10 mg of LDP was precisely weighed and transferred to a 100 mL volumetric flask containing a certain volume of LOS (IS) to obtain a final IS concentration of 2000 ng/mL. About 70 mL of methanol was added and the mixture was shaken vigorously for 15 min, followed by sonication for 30 min to ensure complete extraction. The solution was then diluted to volume with the mobile phase (MP) to obtain a concentration of 100 µg/mL and filtered through a 0.45 μm membrane filter. Further dilution with MP produced a working solution of 10 µg/mL.

#### Remdesivir, Moxifloxacin hydrochloride and levodropropizine (co-formulated)

The fixed-dose combination was prepared since RDV, MFX, and LDP fixed-dose formulations were not readily available. The laboratory-prepared mixture was designed to simulate co-administered pharmaceutical products based on their labeled doses and therapeutic use. The excipients were selected based on the labeled composition of the corresponding marketed formulations of each drug. Tussistop tablets (60 mg/tablet) and Moxiflox tablets (400 mg/tablet) were finely powdered and mixed with four Remdesivir-Eva vials (100 mg/20 mL each). An aliquot of this mixture equivalent to 10 mg of RDV, 6 mg of LDP, and 40 mg of MFX was transferred to a 100 mL volumetric flask containing a specified volume of LOS (IS) to obtain a final IS concentration of 2000 ng/mL. About 70 mL of methanol was added, and the mixture was shaken vigorously for 15 min, followed by sonication for 30 min to ensure complete extraction. The solution was then diluted to volume with the mobile phase (MP) and filtered through a 0.45 μm membrane filter, yielding stock concentrations of 100 µg/mL RDV, 60 µg/mL LDP, and 400 µg/mL MFX. Appropriate dilution with MP was performed to obtain final working concentrations of 10 µg/mL RDV, 6 µg/mL LDP, and 40 µg/mL MFX.The composition of the pharmaceutical mixture containing RDV, MFX, and LDP was determined using the corresponding regression equations.

No interference from common pharmaceutical excipients present in the analyzed dosage forms was observed at the retention times of the studied analytes, confirming the selectivity of the proposed method for the determination of RDV, MFX, and LDP in their pharmaceutical formulations.

### The reported method

- The published procedure for RDV uses an HPLC method using a Reversed-phase Agilent C18 column and water acidified with orthophosphoric acid (A) (pH 4). The gradient elution was performed using acetonitrile (B). The A: B ratio remained at 70:30 by volume from 0 to 4 min. The run was completed at this ratio until the end of the period, after the mobile phase changed linearly until it reached A: B (45:55 by volume) at 6 min. The flow rate was 1 mL/min, and the UV detection wavelength was 240 nm^61^.
- The reported method for MFX is an HPLC procedure with a Kromasil 100-5C18 column and a mobile phase that contains 0.05% trifluoracetic acid (38:62 by volume) and methanol. 290 nm was the UV detection wavelength, while 1.1 mL/min was the flow rate^62^.
- The HPLC method for LDP that has been published uses an Inertsil C18 column and a mobile phase that contains acetonitrile as well as 0.1% triethylamine in water (50:50 by volume) at pH 3. One milliliter per minute was the flow rate, and the UV detection wavelength was 240 nm^44^.

Comparison with previously reported methods reveals that the proposed method offers several advantages. Unlike the reported HPLC methods, which rely on organic solvents such as acetonitrile or methanol, the current method utilizes a micellar mobile phase, significantly reducing environmental impact. Moreover, the proposed method enables simultaneous determination of RDV, MFX, and LDP with higher sensitivity due to fluorescence detection, while previously reported methods are limited to single or fewer analytes with UV detection. In addition, the applicability of the method to plasma samples further highlights its suitability for bioanalytical purposes. A detailed comparison is presented in Tables 9 and 1 SI.

**Table 1:** Chromatographic conditions optimized for the determination of the studied drugs using the proposed method.

Parameter	Number of theoretical plates				Resolution				Tailing factor			
	RDV	MFX	LDP	LOS	RDV	MFX	LDP	LOS	RDV	MFX	LDP	LOS
SLS concentration (M)												
0.05 0.1 0.15 0.2	2024	3560	3231	2462	4.16	2.07	9.22	-	1.19	1.65	1.14	0.88
	2116	3938	3556	2647	4.37	2.13	9.94	-	1.07	1.48	1.09	0.91
	1931	3168	3184	2359	3.57	1.92	8.98	-	1.22	1.73	1.25	0.85
	1865	2801	2954	2107	3.33	1.87	8.14	-	1.28	1.79	1.27	0.83
Brij-35 concentration (M)												
0.02	1851	2917	2146	1930	3.19	1.79	6.37	-	1.15	1.52	1.17	0.90
0.03	2116	3938	3556	2647	4.37	2.13	9.94	-	1.07	1.48	1.09	0.91
0.04	1987	3040	2293	2281	3.82	1.95	7.61	-	1.24	1.62	1.19	0.86
Flow rate												
0.5 ml/min	1541	2623	2525	1985	3.84	1.92	8.13	-	1.21	1.62	1.27	0.87
1 ml/min	2116	3938	3556	2647	4.37	2.13	9.94	-	1.07	1.48	1.09	0.91
PH												
4	2089	3017	2043	1934	2.24	1.83	10.96	-	1.08	0.81	1.02	1.23
6	2116	3938	3556	2647	4.37	2.13	9.94	-	1.07	1.48	1.09	0.91
8	1954	2842	1566	2369	6.39	1.91	5.72	-	1.69	1.21	1.15	0.85

### Application to spiked plasma

To 10 mL centrifuge tubes that containing 1 mL of drug-free plasma, different aliquots of RDV, MFX, and LDP standard solutions (10 µg/mL) containing a specific volume of LOS were pipetted. The protein is then denatured by adding 3 mL of methanol. A vortex shaker was used to mix the contents of the centrifuge tubes, and then the tubes were centrifuged for 30 min at 4000 rpm. The resulting supernatant was carefully separated to minimize any residual particulate matter. After being dried out with a rotary evaporator under vacuum at a controlled temperature not exceeding 40 °C to avoid analyte degradation, the protein-free supernatants were reconstituted in methanol, moved to 10-mL volumetric flasks, and finally the volume was increased to 10 mL using MP resulting in a final solvent composition predominantly matching the mobile phase to ensure optimal peak shape and retention behavior. The entire procedure was carried out again for mixed samples containing all studied drugs across the working concentration ranges. We calculated the RDV, MFX, and LDP contents using the proper regression equation. To further ensure method reliability in biological matrix, selectivity was confirmed by analyzing blank plasma samples and no endogenous interference was observed at the retention times of the analytes.

## Results and discussion

**Fig. 2:**
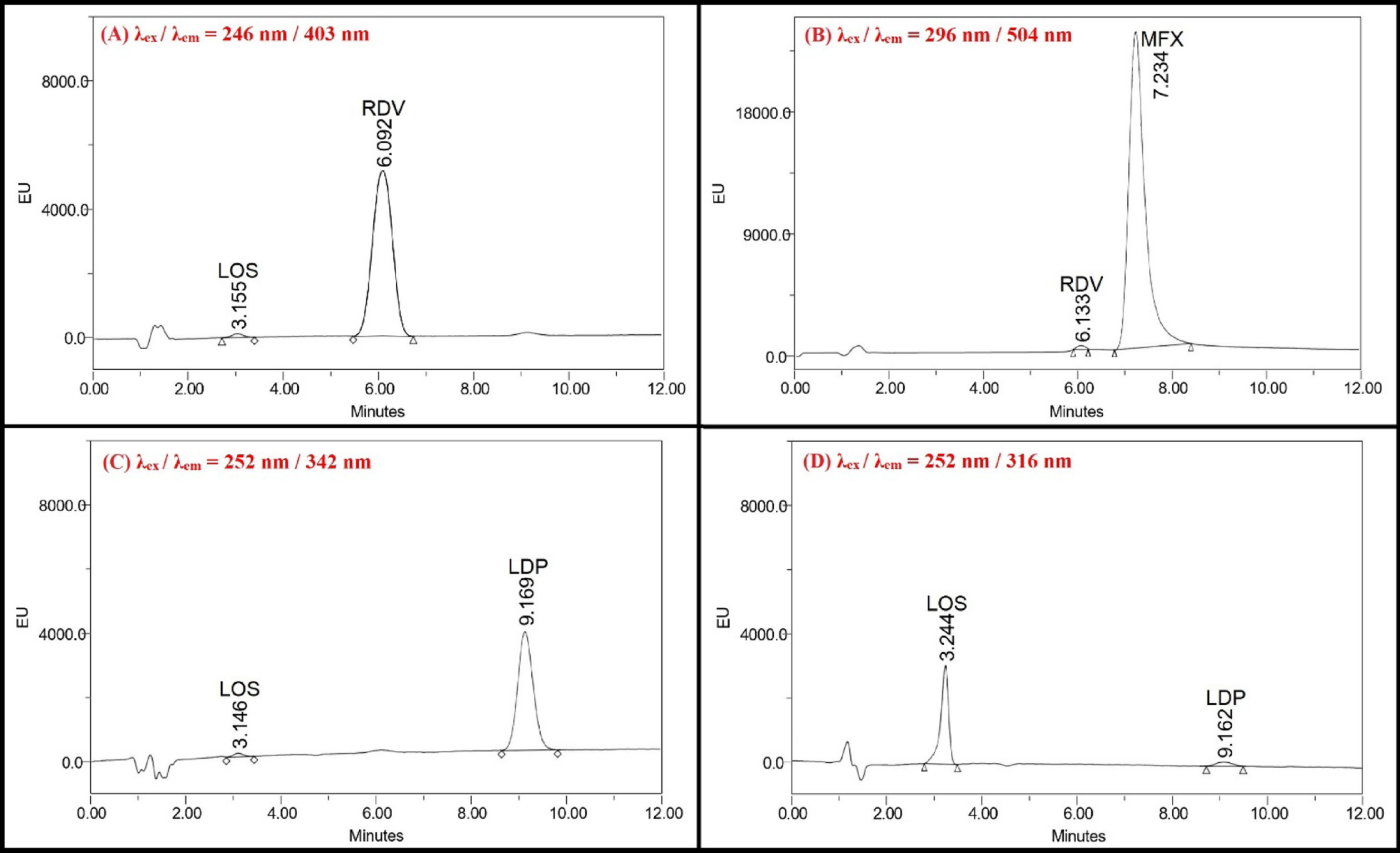
HPLC chromatogram of RDV (2000 ng/mL), MFX (2000 ng/mL), and LDP (4000 ng/mL) in their pure forms containing internal standard LOS (2000 ng/mL), where (**A**) RDV, (**B**) MFX, (**C**) LDP, and (**D**) LOS.

The simultaneous analysis of RDV, MFX, and LDP in bulk, various dose forms, and spiked human plasma was confirmed using a micellar liquid chromatographic approach that is environmentally friendly, simple, accurate, sensitive, and selective. In this method, analysis of the three drugs at the same run in time less than twelve minutes using the micellar isocratic elution technique, validating its ease of use from a chromatographic standpoint, especially in plasma samples due to the method’s capacity to detect low concentrations of RDV, MFX, and LDP below and around their C_max_ using losartan as internal standard. Figure 2 depicts well-resolved symmetrical peaks for RDV, MFX, LDP and LOS acquired at 6.1, 7.2, 9.2 and 3.2 min, respectively. This method was utilized to quickly examine the various pharmaceutical formulations of the researched drugs, as well as spiked human plasma.

### Development and optimization of the approach

The main objective is to separate or resolve the three drugs as best as possible in a short amount of time and with minimal environmental damage. Numerous factors, such as the column selection, pH, flow rate, mobile phase composition, and excitation and emission wavelengths for RDV, MFX, and LDP, were examined in order to optimize the HPLC process.

#### Column selection

Two types of columns were examined: Waters Symmetry C18 (75 × 4.6 mm, 5 μm) and Hypersil BDS C18 (150 × 4.6 mm, 5 μm). Symmetry C18 was ineffective for the separation due to poor separation and peak broadening as evidenced by lower resolution, reduced theoretical plate count, and noticeable peak asymmetry. On the other hand, the Hypersil BDS C18 column has a number of advantages, such as a shorter elution time and better resolution as confirmed by the obtained system suitability parameters, where resolution values exceeded 2, tailing factor was < 2, and column efficiency (N) was > 2000, as presented in Table 6. This improved performance can be attributed to the base-deactivated silica (BDS) surface, which minimizes secondary interactions with residual silanol groups, resulting in enhanced peak symmetry and separation efficiency. The Hypersil BDS C18 column was kept at a steady 40 °C temperature.

#### Optimization of mobile phase

Micellar eluent systems provide various advantages, including low prices, high elution power, minimal environmental effect, and safe disposal^52^. As a result, micellar eluent systems alone were used in this work. Several adjustments to the composition and ratios of the eluent system were made to improve the performance of the chromatographic system while having a quick elution time and minimal environmental impact.

Two factors were present in the micellar eluent systems under investigation: pH and surfactant concentrations.

##### Surfactants’ concentrations

SLS and Brij-35 were the surfactants employed in this attempt. The stationary phase’s polarity was enhanced by the SLS, which directed the sulfate groups outside and bound its monomers to the hydrophobic areas. Experiments revealed that utilizing SLS alone with the buffer resulted in peak broadening and poor resolution, which were resolved by adding Brij-35. Brij-35 was employed to increase resolution because it formed H-bonds with the three medications at different strengths through its oxyethylene chains, which are outward-oriented chains brought on by Brij-35 binding to the stationary phase^52^. Consequently, experiments were conducted with 0.1 M SLS combined with different Brij-35 concentrations between 0.02 M and 0.04 M and 0.03 M Brij-35 combined with different SLS concentrations between 0.05 M and 0.2 M. For SLS and Brij-35, the ideal concentrations were 0.1 M and 0.03 M, respectively. As demonstrated in Table 1, concentrations above 0.1 M led to poor resolution and peak asymmetry, as evidenced by a notable drop in theoretical plate resolution of the peaks, whereas concentrations below 0.1 M produced prolonged retention periods for SLS. Peak asymmetry was observed for Brij-35 at concentrations greater than 0.03 M, while poor separation, lengthy retention periods, and peak asymmetry were observed at concentrations below 0.03 M.

##### PH

During the method development, orthophosphoric acid and NaOH were used to adjust pH levels ranging from 4 to 8 to improve separation of three drugs. While keeping the mobile phase’s composition and flow rate constant, the impact of changing the mobile phase’s pH on the developed method’s chromatographic performance was investigated. It was demonstrated that when the pH was shifted below or above 6, the three drugs’ sensitivity declined as indicated by reduced peak areas and lower signal response, in addition to deterioration in resolution, tailing factor, and number of theoretical plates, as shown in Table 1. This behavior can be explained based on the pKa values of the studied analytes, where changes in pH affect their degree of ionization and consequently their interaction with both the stationary and micellar mobile phases. At pH 6, a balanced ionization state is achieved, leading to optimal retention and separation. Moreover, the phosphate buffer system (0.02 M sodium dihydrogen phosphate) at pH 6 provides sufficient buffering capacity to maintain pH stability throughout the analysis, ensuring consistent chromatographic performance. It was found that a pH of 6 was ideal.

##### Flow rate

The impact of the mobile phase flow rate on the pharmaceuticals under study’s chromatographic process was confirmed; a flow rate of 1 mL/min was found to be an effective chromatographic process in a fair amount of time. As demonstrated in Table 1, a lower flow rate of 0.5 mL/min produced wider peaks, as evidenced by fewer theoretical plates and resolution with a longer analytical run time.

#### Detection wavelength

The benefit of the Waters 2475 fluorescence detector was its ability to detect at a variety of wavelengths, which improved selectivity. Without the MFX and LDP peak showing up, two peaks at λ exi/λ emi = 246/403 nm emerged at 3.155 and 6.092 min, representing LOS and RDV, respectively. In the absence of the LOS and LDP peak, two peaks at λexi /λ emi = 296/504 nm, representing RDV and MFX, respectively, emerged at 6.133 and 7.234 min. It was not sensitive enough to achieve the RDV plasma concentration, and the RDV peak area at these wavelengths did not respond linearly. Without the development of the RDV and MFX peak, two peaks representing LOS and LDP, respectively, emerged at 3.146 and 9.169 min at λ exi /λ emi = 252/342 nm. Without the RDV and MFX peak showing up, two peaks representing LOS and LDP, respectively, emerged at 3.244 and 9.162 min at λ exi /λ emi = 252/316 nm. For RDV, MFX, LDP, and LOS, measurements were made at four distinct excitation wavelengths (246, 296, 252, and 252 nm) and four emission wavelengths (403, 504, 342, and 316 nm), respectively. In complex biological matrices, this led to analysis with high sensitivity, broad linear ranges, and selectivity.

### Validation of the method

The recommended analytical method was validated in terms of particular elements such as the following, per ICH :^63^.

#### Linearity

To ascertain the linearity ranges for the three mentioned medications, the response is compared to the corresponding concentration using the ratio of (peak area of analyte to the IS). Seven distinct concentrations ranging from 80 to 2000 ng/mL for both RDV and MFX and from 160 to 4000 ng/mL for pure LDP were used to assess the linearity of the approach. Since the linearity ranges in plasma of the suggested approach included the C_max_ for all of the medicines mentioned, it could be applied to the plasma of actual patients. The C_max_ values for RDV^64^, MFX^65^, and LDP^66^ were, respectively, 57.5–4420 ng/mL, 3560 ng/mL, and (261.22–325.46) ng/mL. Table 2 displayed the parameters for the regression equations. The regression equations were characterized by high correlation coefficients (r² ≥ 0.9997), and the small intercept values indicate minimal systematic error. The linearity was further supported by the close agreement between calculated and nominal concentrations, confirming the absence of significant deviation from linearity. Calibration curves were constructed by plotting the peak area ratios of analytes to internal standard versus concentration. The method showed excellent linearity over the studied ranges with high correlation coefficients. The corresponding calibration plots are provided as supplementary Figs. 1SI–3SI to visually confirm linearity.

**Table 2:** Regression and validation parameters for the determination of RDV, MFX, and LDP using the proposed HPLC method.

Parameters	RDV	MFX	LDP
Linearity range (ng/mL)	80-2000	80-2000	160–4000
Slope	0.0265	0.2144	0.0067
Intercept	− 0.8455	8.2177	− 0.3632
LOD (ng/mL)	24.75	26.39	51.23
LOQ (ng/mL)	75.02	79.98	155.25
Coefficient of determination (r^2^)	0.9997	0.9997	0.9997
Accuracy (% R) ^a^	100.16	101.17	99.16
Precision (% RSD) ^b^			
Repeatability	1.355	0.533	1.710
Intermediate precision	0.383	1.262	0.109
Robustness (% R ^c^ ±% RSD)			
Flow rate (± 0.02 ml/min)	101.85 ± 1.178	99.62 ± 1.953	100.61 ± 0.779
pH (± 0.2)	100.67 ± 0.242	100.68 ± 1.529	102.40 ± 1.288
Temperature (± 2 °C)	101.54 ± 1.706	101.49 ± 1.3708	100.56 ± 0.833
SLS concentration (± 0.02 M)	101.79 ± 1.067	101.76 ± 1.833	100.64 ± 1.590

^a^Nine determinations on average (three concentrations repeated three times).

^b^ %RSD of nine measurements (three concentrations, three repetitions).

^c^ Average of 3 determinations.

#### LOD and LOQ

The sensitivity of the proposed approaches was assessed through the calculation of LOD and LOQ (Table 2). The outcomes in Table 2 indicate the high sensitivity of the proposed method, as evidenced by the low LOD and LOQ values enabling accurate detection and quantification of the analytes at low concentration levels. LOD and LOQ were calculated based on the standard deviation of the response and the slope of the calibration curve, according to ICH Q2(R1) guidelines.

#### Accuracy and precision

The proposed method was used to find three concentration levels in triplicate that were in the linearity ranges of the three drugs: RDV (800, 1200, and 1600 ng/mL), MFX (800, 1200, and 1600 ng/mL), and LDP (1600, 2400, and 3200 ng/mL). We used the proposed method to find the %RSD of three concentration levels that covered the linearity range of each drug (800, 1200, and 1600 ng/mL for RDV, 800, 1200, and 1600 ng/mL for MFX, and 1600, 2400, and 3200 ng/mL for LDP) within one day for repeatability and on three consecutive days for intermediate precision. The high %R in Table 2 shows that the suggested method is accurate. Table 2 also shows that the method is very accurate because its %RSD values are very small. The obtained accuracy and precision results comply with the accepted bioanalytical criteria, where % recovery falls within ± 15% (± 20% at LLOQ) and %RSD does not exceed 15%, confirming the reliability of the proposed method.

#### Specificity

**Fig. 3:**
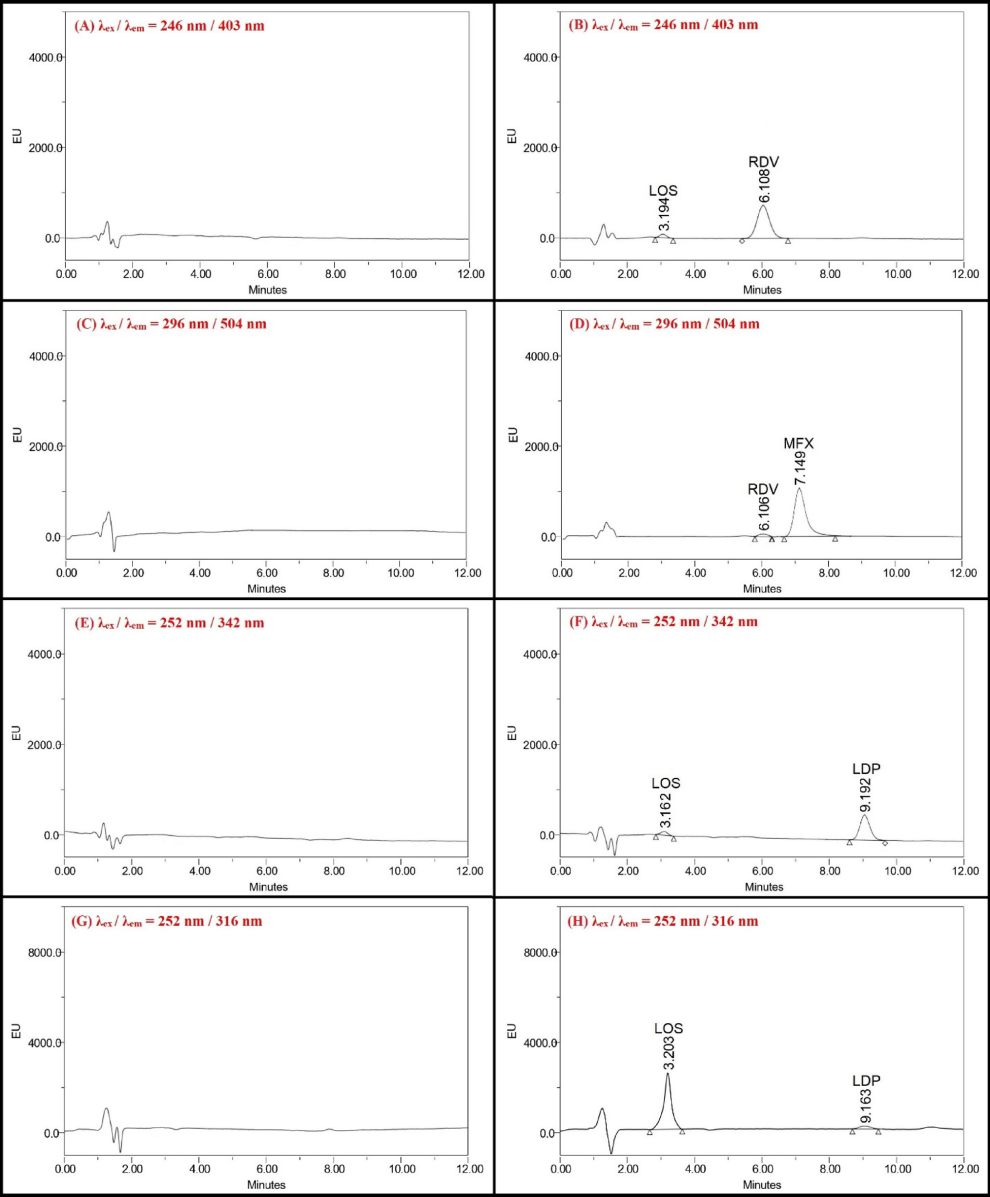
HPLC chromatograms showing selectivity of the proposed method in human plasma, where (**A**) blank plasma and (**B**) plasma spiked with RDV, (**C**) blank plasma and (**D**) plasma spiked with MFX, (**E**) blank plasma and (**F**) plasma spiked with LDP, and (**G**) blank plasma and (**H**) plasma spiked with LOS (internal standard) using IS of LOS (2000 ng/mL).

We had to make lab-prepared mixes with different amounts of the three drugs. The mixes were analyzed according to the experimental protocols. Table 3 shows that the results were good and satisfactory. We also looked at how excipients affected drug estimates by using the standard addition method on pharmaceutical samples that had already been tested (Table 4). Table 5 shows that the usual addition method works well to stop excipient interference, which means that the method is selective. It is also very important to make sure that the suggested method works well in plasma. Figure 3 shows the chromatograms of plasma-free samples and samples of plasma that had the three drugs added to them. This shows that the suggested method is selective without being affected by plasma endogenous components. The peaks of the mentioned drugs showed up in the spiked plasma chromatogram at the times they were supposed to, but they weren’t there in the blank plasma chromatogram.

**Table 3:** Determination of RDV, MFX, and LDP in laboratory-prepared mixtures using the proposed HPLC method.

Laboratory prepared mixture (ng/mL)			% Recovery*		
RDV	MFX	LDP	RDV	MFX	LDP
1200	1200	2400	100.45	101.16	98.33
800	1200	3200	98.17	101.44	101.87
800	1600	800	97.69	101.70	101.81
2000	800	800	101.22	100.62	102.83
1600	2000	1600	101.26	98.99	99.23
400	400	800	98.81	98.51	101.81
Mean ± %RSD			99.59 ± 1.581	100.40 ± 1.33	100.97 ± 1.75

^*^ Three determinations averaged.

**Table 4:** RDV, MFX, and LDP determination using the suggested HPLC approach in pharmaceutical formulations and co-formulated dosage forms.

Remdesivir-Eva 100 mg/vial		Moxiflox 400 mg/tablet		Tussistop 60 mg/tablet		Co-formulated dosage form					
						RDV		MFX		LDP	
Conc. (ng/mL)	% R *	Conc. (ng/mL)	% R *	Conc. (ng/mL)	% R *	Conc. (ng/mL)	% R *	Conc. (ng/mL)	% R *	Conc. (ng/mL)	% R *
300	99.81	1200	100.37	180	100.45	300	98.52	1200	98.88	180	101.85
350	98.66	1400	101.28	210	99.36	350	100.59	1400	101.25	210	99.65
400	100.16	1600	99.95	240	101.48	400	99.48	1600	99.18	240	101.28
450	101.32	1800	100.48	270	99.79	450	101.29	1800	101.58	270	98.48
500	99.85	2000	99.15	300	101.04	500	99.39	2000	99.68	300	100.77
Mean	99.96	Mean	100.25	Mean	100.42	Mean	99.85	Mean	100.11	Mean	100.41
%RSD	0.951	%RSD	0.777	%RSD	0.867	%RSD	1.090	%RSD	1.226	%RSD	1.342

^*^ Average of 3 determinations.

**Table 5:** Determination of the studied drugs by the standard addition technique using the proposed HPLC method.

Pharmaceutical (ng/mL)	RDV		MFX		LDP	
	Pure added (ng/mL)	% R *	Pure added (ng/mL)	% R *	Pure added (ng/mL)	% R *
RDV 350 (352.07)*	200	101.68	200	101.48	200	98.62
MFX 1400 (1417.50)*	400	100.52	400	98.88	1000	101.44
LDP 210 (209.27)*	1000	99.98	500	100.09	2000	100.78
Mean		100.72		100.15		100.28
%RSD		0.862		1.299		1.471

^*^ Average of 3 determinations.

#### Robustness

The robustness of the proposed strategy was assessed by looking at how the analytical performance of the recommended technique was affected by minor deliberate modifications to the optimal conditions. The SLS eluent concentration (0.1 ± 0.02 M), pH (± 0.20), flow rate (± 0.02 mL/min), and column temperature (± 2 °C) all showed minor variations. In comparison to the initial (ideal) settings, the deliberate modifications to the previously specified parameters resulted in negligible variations in recovery percentages, demonstrating the durability and dependability of the recommended strategy (Table 2).

### System suitability

To ensure that the eluent system was operating properly, system suitability parameters such as capacity factor, resolution, tailing factor, and column efficiency (the number of theoretical plates) were investigated. Under ideal conditions, the proposed method was appropriate for the separation and quantification of the three drugs, as all of the calculated parameters in Table 6 showed satisfactory performance in comparison with the established acceptance criteria. The resolution (Rs = 2.13–9.94) was higher than 2, the capacity factor (K′ = 5.09–8.14) was within the acceptable range of 1–10, the tailing factor (T = 1.07–1.48) was less than 2, and the theoretical plates (*N* = 2116–3938) exceeded 2000, confirming that all parameters in Table 6 fell within the permitted limits (reference values)^67^.

**Table 6:** System suitability evaluation of the proposed HPLC method for determining RDV, MFX, and LDP.

Parameters	RDV	MFX	LDP	LOS	Reference value^67^
Retention time (t_R_, min)	6.092	7.234	9.169	3.244	–
Resolution (Rs)	4.37	2.13	9.94	-	> 2
Capacity factor (K′)	5.09	6.15	8.14	5.62	1 < K′<10
Tailing factor (T)	1.07	1.48	1.09	0.91	< 2
Column efficiency (N)	2116	3938	3556	2647	> 2000

### Pharmaceutical application

As indicated in Table 4, RDV, LDP, and MFX were calculated as single dosage forms or as laboratory co-formulated dosage forms as part of the suggested method. It was shown that excipients and additives had no effect based on the results of the standard addition technique, which are displayed in Table 5.

### Spiked human plasma

The three medications were quantified in human plasma using the high sensitivity (measured in terms of low LOD values) attained by the suggested HPLC method (Table 7). The findings listed in Table 7 demonstrated that the suggested method can be used to conduct bioequivalence tests of the three medications concurrently without causing any interference from the matrices of biological fluids.

**Table 7:** Determination of RDV, MFX and LDP in spiked human plasma by the proposed HPLC method.

RDV			MFX			LDP		
Added (ng/mL)	Found* (ng/mL)	%Recovery	Added (ng/mL)	Found* (ng/mL)	%Recovery	Added (ng/mL)	Found* (ng/mL)	%Recovery
100	95.08	95.08	100	93.48	93.48	180	171.09	95.05
400	379.28	94.82	400	386.368	96.59	300	288.45	96.15
800	781.20	97.65	800	751.28	93.91	900	860.13	95.57
1200	1140.36	95.03	1200	1141.63	95.14	1800	1738.44	96.58
1800	1728.90	96.05	1800	1707.05	94.84	3600	3437.64	95.49
Mean ± %RSD		95.73 ± 1.229	Mean ± %RSD		94.79 ± 1.277	Mean ± %RSD		95.77 ± 0.626

* Average of 5 determinations.

### Statistical analysis

For the analysis of the three drugs in their pure form, the results of the proposed HPLC method were statistically compared with those of previously reported methods^44,61,62^. The t-test (for accuracy) and F-test (for precision) were applied for each drug, as summarized in Table 8. The calculated t and F values were lower than the corresponding theoretical values at the 95% confidence level, indicating that there is no statistically significant difference between the proposed and reported methods in terms of accuracy and precision. These findings confirm the suitability of the proposed HPLC method for routine quality control analysis of the studied drugs, as demonstrated by the statistical comparison presented in Table 8.

**Table 8:** Statistical analysis and determination of RDV, MFX, and LDP in pharmaceutical preparations by the proposed HPLC and reported methods.

Parameters	RDV		MFX		LDP	
	Proposed method	Reported method^61^	Proposed method	Reported method^62^	Proposed method	Reported method^44^
Number of measurements	5	5	5	5	5	5
Mean % Recovery	99.96	100.05	100.25	99.81	100.42	101.14
% RSD	0.950	1.343	0.777	1.484	0.867	0.930
Variance	0.9035	1.806	0.607	2.195	0.757	0.885
Student’s *t*-test * (2.306)	0.119	——	0.577	——	1.253	——
*F*-value * (6.388)	1.998	——	3.616	——	1.169	——

* The values in parenthesis are tabulated values of “*t*” and “*F*” at (*P* = 0.05).

### Method evaluation of greenness profiles

GAC seeks to advance an environmentally responsible and sustainable method of analytical chemistry that preserves high accuracy and reliability while lessening its impact on the environment. Number of methods, including qualitative, semi-quantitative, and quantitative approaches, have been developed in recent years to assess greenness.

The analytical eco-scale, GAPI and AGREE evaluation techniques were used to evaluate the environmental friendliness of the suggested approach. Interestingly, all of the methods used to measure greenness backed up the notion that it is a very environmentally friendly strategy. Three previously published techniques were compared to the suggested HPLC process^44,61,62^. Based on the three measures employed, the suggested technique had the lowest environmental impact, as shown in Table 9.

- *Greenness is evaluated using the analytical eco-scale*. A recently created all-inclusive, semi-quantitative tool for assessing an approach’s greenness^59^. It is based on subtracting points, sometimes called penalty points, for aspects of the analytical process that do not align with the 12 GAC concepts. The score is higher (nearer 100) for an analysis that is more ecologically friendly. In Table 9, the penalty points for the suggested methodology totaled 15 points, resulting in a total score of 85, reflecting the new method’s higher greenness compared to the reported methods of RDV and MFX with total scores 77 and 73, respectively. But it is nearly the same greenness as LDP reported method.
- *Greenness evaluation in accordance with GAPI.* While GAPI allows for a more comprehensive semi-quantitative analysis, analytical eco-scale allows for a more basic evaluation of greenness. This measure is reliable because it provides an ecological evaluation of the entire analytical process, including sample collection, preservation, transportation, and preparation to the final analysis site. It was composed of five pentagrams, each representing fifteen distinct aspects. There are fifteen parts to this colored pentagram^68^. The section may be green, yellow, or red, depending on how green it is. Only three red pentagrams were seen for the developed method, while the majority of pentagrams were green and yellow, according to the GAPI metric. These red zones are mainly attributed to unavoidable steps such as protein precipitation using a limited volume of methanol, which was required to achieve adequate plasma deproteinization and acceptable analyte recovery. Although the micellar system significantly reduces the consumption of hazardous organic solvents in the mobile phase, complete elimination of organic solvent from the sample preparation step was not feasible without compromising extraction efficiency and sensitivity. Also, the suggested technique’s greenness is demonstrated by the five green zones in its GAPI pictograms (Table 9), as opposed to the three green zones in the RDV and MFX methods that were reported.
- *Greenness evaluation in accordance with AGREE.* The greenness of the developed and reported methods was evaluated using the Analytical Greenness (AGREE) metric, a comprehensive tool that assesses the environmental sustainability of analytical procedures based on the 12 principles of Green Analytical Chemistry and expresses the result as a single score ranging from 0 (least green) to 1 (most green)^69^.

The proposed method achieved the highest AGREE score (0.66), indicating superior environmental performance compared to the reported methods, which showed lower scores of 0.59 for the levodropropizine method, 0.54 for the remdesivir method, and 0.47 for the moxifloxacin method as displayed in Table 9. The relatively lower scores of the reported methods can be attributed to their reliance on hazardous organic solvents, multi-step sample preparation procedures, and higher energy consumption, particularly in methods involving biological matrices.

**Table 9:** Evaluation of the greenness of the proposed and reported HPLC methods using Eco-Scale, GAPI and AGREE.

Eco-scale method				
Parameters	Proposed HPLC method	Reported method for RDV^61^	Reported method for MFX^62^	Reported method for LDP^44^
Reagents				
Methanol	6	6	12	-
Orthophosphoric acid	2	2	-	-
Acetonitrile	-	8	-	4
SLS	0	-	-	-
Brij-35	0	-	-	-
Trifloroacetic acid	-	-	8	-
Sodium dihydrogen phosphate	0	-	-	-
Triethylamine	-	-	-	6
Water	-	0	-	-
Instruments				
Spectrofluorometer/ /HPLC				
Energy	1 [> 0.1 kWh/sample]	1 [> 0.1 kWh/sample]	1 [> 0.1 kWh/sample]	1 [> 0.1 kWh/sample]
Occupational hazard	0	0	0	0
Waste	6	6	6	3
Total penalty points	Σ 15	Σ 23	Σ 27	Σ 14
Analytical eco-scale total score	85	77	73	86
Analytical eco-scale total score^a, b^	Excellent green analysis	Excellent green analysis	Acceptable green analysis	Excellent green analysis
GAPI method				
GAPI pentagram				
AGREE				

^a^ Analytical eco-scale total score = 100- total penalty points.

^b^ If the score is greater than 75, indicates that the green analysis is excellent.

If the score is greater than 50, it indicates that the green analysis is acceptable.

If the score is of 50 or less, it indicates insufficient green analysis.

In contrast, the proposed method demonstrates improved greenness due to the use of a micellar mobile phase that reduces solvent toxicity and environmental impact. Although some sample preparation steps are still required, the overall method provides a more sustainable and environmentally friendly alternative for routine analysis.

## Conclusion

In order to determine remdesivir, levodropropizine, and moxifloxacin hydrochloride simultaneously in their pure forms, combination dosage forms, and in human plasma containing these medications, we have successfully developed a sensitive and environmentally friendly micellar HPLC method with fluorescence detection. The method achieved excellent sensitivity, high resolution, and rapid analysis devoid of dangerous organic solvents. Using losartan as an internal standard enhanced the accuracy of quantification in complex matrices. This work showed a great adherence to the fundamentals of green analytical chemistry given the outstanding score on the Analytical Eco-Scale and GAPI. The method’s sufficiency for routine pharmaceutical analysis and therapeutic drug monitoring was verified and deemed acceptably reliable. Therefore, this work is indicative of the developing sustainable analytical methods targeted to COVID-19 therapeutics.

## Supplementary Information

Below is the link to the electronic supplementary material.

Supplementary Material 1

## Data Availability

The data that support the findings of this study are available from the corresponding author, upon reasonable request.
