# Supplementary material for: Development and validation of HPLC-FLD method for simultaneous determination of ternary therapy used for COVID-19 in plasma samples
Source: Sci Rep. 2026 Jun 16;16:18696. doi: 10.1038/s41598-026-53885-7 (PMC13272654; doi:10.1038/s41598-026-53885-7)
Supplement: Supplementary file 1 — Supplementary Material 1 [file 41598_2026_53885_MOESM1_ESM.docx]

**Development and validation of HPLC-FLD method for simultaneous determination of ternary therapy used for COVID-19 in plasma samples**

**Eman A. Madbouly^1,*^, Abdalla A. El-Shanawani ^1^, Sobhy M. El-Adl^1^,** **Ahmed S. Abdelkhalek^1^**

^1^ *Department of Medicinal Chemistry, Faculty of Pharmacy, Zagazig University, Zagazig, Egypt.*

**SUPPORTING INFORMATION**

**Corresponding authors:**

Eman A. Madbouly: [dr.eman_ashraf@yahoo.com](mailto:dr.eman_ashraf@yahoo.com), [EAMadbouli@pharmacy.zu.edu.eg](mailto:EAMadbouli@pharmacy.zu.edu.eg)

Ahmed S. Abdelkhalek: [ashilal@pharmacy.zu.edu.eg](mailto:ashilal@pharmacy.zu.edu.eg)


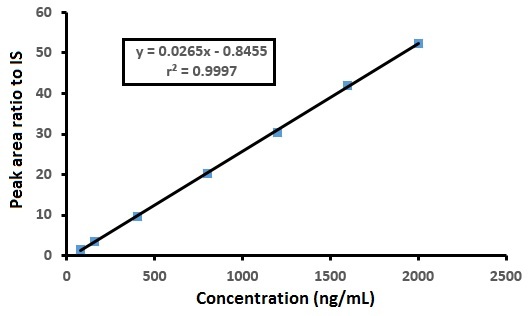


**Figure 1 SI. Calibration curve of RDV by the proposed HPLC method.**

**
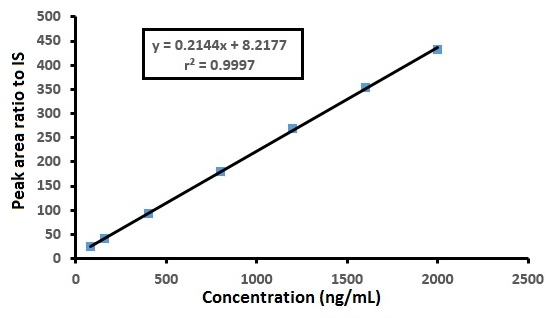
**

**Figure 2 SI. Calibration curve of MFX by the proposed HPLC method.**

**
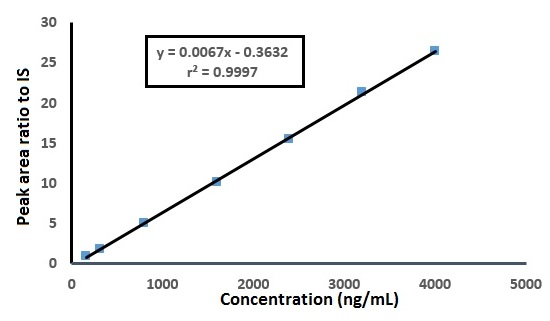
**

**Figure 3 SI. Calibration curve of LDP by the proposed HPLC method.**

**Table 1 SI**

**Comparison of the Proposed HPLC Method with Previously Reported Methods**

| **Drug** | **Method** | **LOD (ng/mL)** | **Run time** |
| --- | --- | --- | --- |
| **RDV** | **Reported HPLC [61]** | **20 ng/mL** | **~10 min** |
| **MFX** | **Reported HPLC [62]** | **16 ng/mL** | **~ 12 min** |
| **LDP** | **Reported HPLC [44]** | **502 ng/mL** | **6 min** |
| **Proposed method** | **Micellar HPLC-FLD** | **24.75 ng/mL (RDV)**  **26.39 ng/mL (MFX)**  **51.23 ng/mL (LDP)** | **< 12 min** |
